# Fabrication and Characterization of Capacitive Micromachined Ultrasonic Transducers with Low-Temperature Wafer Direct Bonding

**DOI:** 10.3390/mi7120226

**Published:** 2016-12-11

**Authors:** Xiaoqing Wang, Yude Yu, Jin Ning

**Affiliations:** 1 State Key Laboratory on Integrated Optoelectronics, Institute of Semiconductors, Chinese Academy of Sciences, Beijing 100083, China; xiaoqingwang@semi.ac.cn; 2 University of Chinese Academy of Sciences, Beijing 100049, China; 3 Engineering Research Center for Semiconductor Integrated Technology, Institute of Semiconductors, Chinese Academy of Sciences, Beijing 100083, China; ningjin@semi.ac.cn

**Keywords:** capacitive micromachined ultrasonic transducers (CMUTs), low temperature wafer direct bonding, contact angle, surface roughness

## Abstract

This paper presents a fabrication method of capacitive micromachined ultrasonic transducers (CMUTs) by wafer direct bonding, which utilizes both the wet chemical and O_2_ plasma activation processes to decrease the bonding temperature to 400 °C. Two key surface properties, the contact angle and surface roughness, are studied in relation to the activation processes, respectively. By optimizing the surface activation parameters, a surface roughness of 0.274 nm and a contact angle of 0° are achieved. The infrared images and static deflection of devices are assessed to prove the good bonding effect. CMUTs having silicon membranes with a radius of 60 μm and a thickness of 2 μm are fabricated. Device properties have been characterized by electrical and acoustic measurements to verify their functionality and thus to validate this low-temperature process. A resonant frequency of 2.06 MHz is obtained by the frequency response measurements. The electrical insertion loss and acoustic signal have been evaluated. This study demonstrates that the CMUT devices can be fabricated by low-temperature wafer direct bonding, which makes it possible to integrate them directly on top of integrated circuit (IC) substrates.

## 1. Introduction

Ultrasonic transducers are widely used in medical imaging, gas-flow metering, industrial cleaning and non-destructive testing. In many areas of application, capacitive micromachined ultrasonic transducers (CMUTs) have been considered an attractive alternative to conventional piezoelectric transducers [1,2,3]. CMUTs can be batch produced with photolithography and other mature techniques in semiconductor industries, which is difficult with piezoelectric transducers [4]. Therefore, CMUTs have a great potential for monolithic integration, which presents multiple benefits such as a reduced system footprint, lower interconnection parasitic capacitance and simplified packing. Moreover, CMUTs provide advantages in size reduction and easier transducer array fabrication [5].

Considerable research on CMUTs has been conducted in recent decades [6,7,8]. To produce such a CMUT device, two main fabrication methods are currently adopted: surface micromachining [9] or wafer bonding [10]. Surface micromachining CMUTs is a technique where the cavity underneath the membrane is created by depositing a sacrificial layer on the substrate. After the membrane deposition, the sacrificial layer is removed with etchant [11]. However, the surface micromachining process sets limitations on the cavity and membrane size because of sticking problems that occur during the releasing step. Moreover, releasing the membranes is difficult due to large capillary forces and may introduce contamination in the cavity, which decreases the success rate of the device [12].

In the wafer bonding process, membranes are fabricated by utilizing silicon on insulator (SOI) wafers or thin silicon wafers [13]. This process adds many design flexibilities and also offers wide material choices. Because the membrane and the cavity are made on separate wafers, it is possible to optimize them both simultaneously. The technique reduces the number of process steps, which increases the control of the fabrication procedure. In addition, there are no wet-release processes to limit the size of the membranes, allowing the fabrication of CMUTs for a wide range of frequencies [13,14]. Nevertheless, conventional wafer bonding technology requires high temperature (>1000 °C) to ensure good bonding quality, which prohibits monolithic integration of CMUTs [15]. Additionally, a high temperature could result in the degradation of thermally sensitive devices and residual stress in processed silicon wafers with different thermal expansion coefficients [16]. To avoid the problems mentioned above, low-temperature wafer bonding which is limited to 400 °C is strongly desirable. Many efforts have been made to reduce the bonding temperature. One approach is to deposit the adhesion material such as titanium and gold, which migrates into silicon at low temperatures [17]. However, this method could bring pollution caused by the adhesion material. Another approach to decrease the temperature is to employ low-temperature wafer direct bonding technology, which is enabled by using activation processes of the surfaces. Such a process consists of wet chemical activation or O_2_ plasma treatment prior to bonding [18,19]. With these surface activation techniques, less energy is required during the thermal annealing steps and they achieve good bonding quality at low temperatures.

In this paper, we report a method to fabricate CMUTs with low-temperature wafer direct bonding at 400 °C. The wet chemical and O_2_ plasma activation processes are employed together. The influences on the surface roughness and water contact angle of the activation treatments are investigated. The low-temperature CMUT fabrication process has been presented in detail. Both electrical and acoustic characteristics of the fabricated devices are measured and discussed.

## 2. Surface Properties by Activation Processes

### 2.1. Contact Angle

The main mechanism of wafer direct bonding at low temperature is to activate the surfaces by creating more hydroxyl (OH) groups, which results in higher hydrophilicity. Two methods of surface activation are utilized: wet chemical and O_2_ plasma activations. The wet chemical activation is usually achieved by treating pre-bonding wafers with various reagent combinations to activate the surfaces. O_2_ plasma activation is a process of applying O_2_ plasma treatment to improve the hydrophilicity of the surfaces.

**Figure 1:**
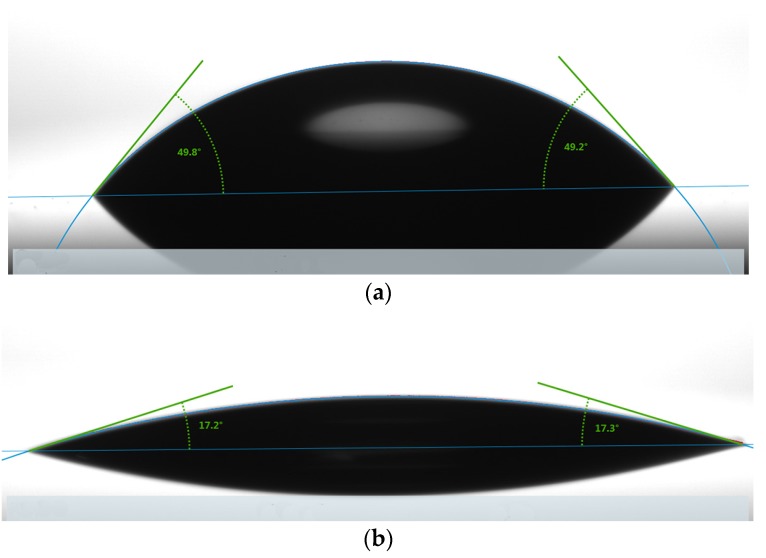
(**a**) The contact angle of as-received wafer; (**b**) The contact angle with the wet chemical activation process.

To investigate the surface hydrophilicity of wafers in practical activation conditions, we measured the contact angle by a drop shape analysis system (DSA100, KRÜSS, Hamburg, Germany). The contact angle is critical to evaluating the hydrophilicity of the surfaces and a lower contact angle means a higher hydrophilicity, which could improve the bonding effect of micro-electromechanical systems (MEMS) and system integration. We measured the contact angles immediately after the wet and oxygen treatments. If the wafers were stored in a normal clean room environment, the contact angle showed no obvious change when the elapsed time was less than 30 min after the wet and oxygen treatment. After 30 min, the contact angle changed significantly over time. The contact angle of as-received Si covered with SiO_2_ without any treatment is 49.5°, as shown in Figure 1a. The RCA clean is a procedure developed by Werner Kern at RCA (Radio Corporation of America) laboratories for processing wafers in the late 1960’s. Then, the wafer was cleaned by H_2_SO_4_/H_2_O_2_ (H_2_SO_4_:H_2_O_2_ = 3:1), a modified RCA1 (H_2_O:H_2_O_2_:NH_4_OH = 5:1:0.2), a modified RCA2 (H_2_O:H_2_O_2_:HCl = 5:1:0.2) and HNO_3_/H_2_O/HF (HNO_3_:H_2_O:HF = 1:20:2 × 10^−4^) solutions. The contact angle is 17.3°, as shown in Figure 1b. It is observed that the contact angle is obviously lower than that of the as-received wafer, which demonstrates the wet chemical activation could improve the hydrophilicity of surfaces.

**Figure 2:**
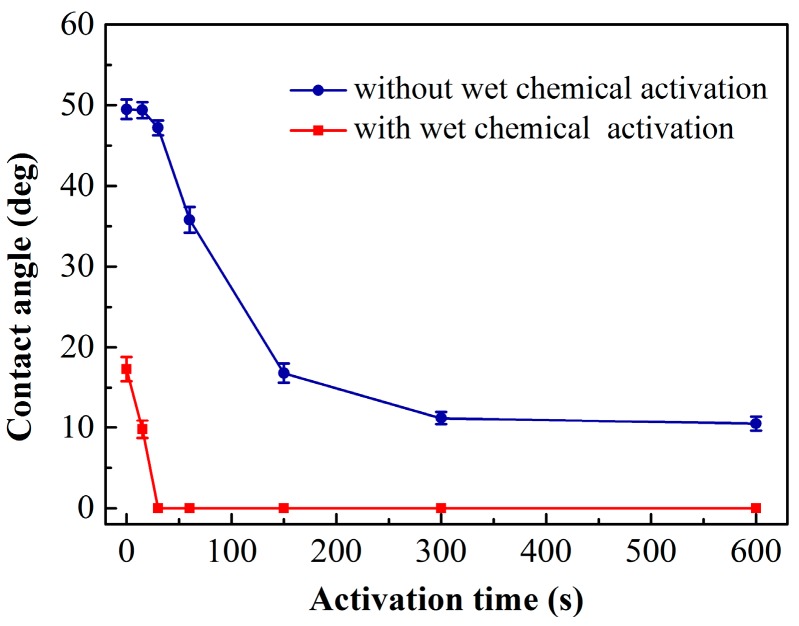
Contact angle as a function of O_2_ plasma activation time.

In order to analyze the effects of O_2_ plasma activation, we measured two sets of wafers. One set was only activated with O_2_ plasma. The other set was processed with wet chemical and O_2_ plasma activations at the same time. The different plasma activation times selected were 15, 30, 60, 150, 300 and 600 s. The contact angles after O_2_ plasma activation for different times are shown in Figure 2. The experimental error is ±1.6°. For the wafer treated only with O_2_ plasma, the contact angle drops with the increase of the activation time. When the activation time is less than 30 s, the contact angle is slightly decreased. Then, the contact angle decreases significantly as the activation time increases from 30 to 300 s. After 300 s, the decrease of the contact angle is not obvious. This may be explained by the O_2_ plasma removing the organic contaminants and reacting with the surface very little during a short activation time. At a higher activation time, O_2_ plasma starts to react with the surface and create more dangling bonds. When the activation time is higher than 300 s, the dangling bonds on the surface become saturated and the contact angle decreases slowly. For the wafer treated with both wet chemical and O_2_ plasma activations, the contact angle decreases with the increase of the activation time immediately. At 30 s, the contact angle is 0° and there is no change with the increase of the activation time. These results indicate that activating the surfaces with wet chemical and O_2_ plasma processes simultaneously can result in better hydrophilicity, which could obtain a good bonding quality in device fabrication.

### 2.2. Surface Roughness

**Figure 3:**
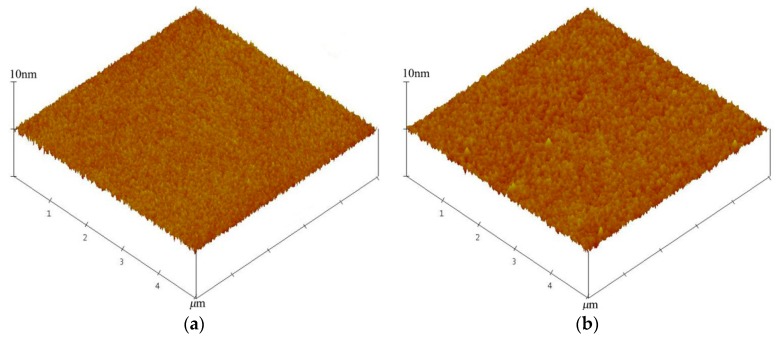
Three-dimensional (3D) atomic force microscopy (AFM) images of wafer surfaces. (**a**) Root mean square (RMS) of as-received wafer is 0.199 nm; (**b**) RMS after etching for cavity is 0.293 nm.

**Figure 4:**
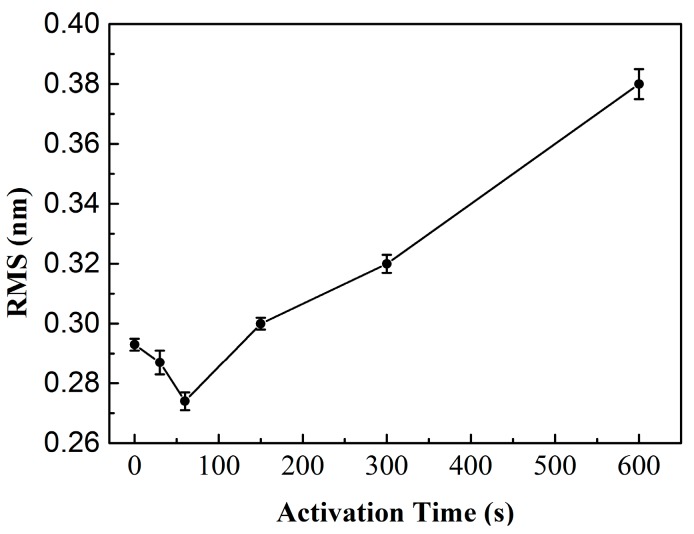
Surface roughness as a function of O_2_ plasma activation time.

When two wafers bond over the entire area, the surface roughness is a key factor to achieving a high bonding quality. Lower surface roughness allows a greater contact area between bonding surfaces. Therefore, surface roughness plays an important role in the adhesion quality in CMUT devices. In our work, the surface roughness was measured using atomic force microscopy (AFM D3100, Veeco, Santa Barbara, CA, USA) in standard tapping mode with a scan area of 5 μm × 5 μm. The root mean square (RMS) method was used to evaluate the surface roughness (*R*_q_). The surface roughness of the as-received Si substrate covered with SiO_2_ is 0.199 nm, as shown in Figure 3a. After the dry etching process for the cavity, the surface roughness increased to 0.293 nm, as shown in Figure 3b. This may be explained by the photoresist and reactive ion etching during the fabrication process possibly roughening the wafer surface. Then, the wafer was treated with O_2_ plasma. The RMS surface roughness of the wafer as a function of activation time is shown in Figure 4. The experimental error is ±0.005 nm. It can be seen that the surface roughness decreases with the increase of the activation time until 60 s, at which time the surface roughness is 0.274 nm. Such a smoothing effect is attributed to the surface cleaning of particles and hydrocarbons by the O_2_ plasma treatment. After an activation time of 60 s, the surface roughness increases significantly with the increasing treatment time. When the O_2_ plasma is used to treat the wafers, it starts etching as well as oxidizing the surfaces. However, the rate of etching is higher than that of oxidation at higher activation times. It indicates that excessive plasma activation could result in more surface damage. The O_2_ plasma activation time of 60 s is suitable for direct bonding due to the lower surface roughness. The benefit is that it reduces the annealing temperature required to achieve a strong bond in the following CMUTs fabrication.

## 3. Low-Temperature CMUT Fabrication Process

Basically, a CMUT cell can be considered as a parallel plate capacitor composed of a membrane suspended above a cavity. When a direct current (DC) voltage is applied between the bottom electrode and the top electrode, the membrane deflects toward the substrate due to the electrostatic force. The electrostatic force is balanced by a restoring mechanical force which is derived from the increased strain in the membrane. The membrane can vibrate to generate the ultrasound by adding an alternating current (AC) voltage on top of the DC bias voltage. Then the mechanical energy is coupled into the surrounding medium. The dimensions and material properties determine the dynamic behavior and frequency characteristics of the CMUT. The specific parameters of a designed structural element are listed in Table 1.

**Figure 5:**
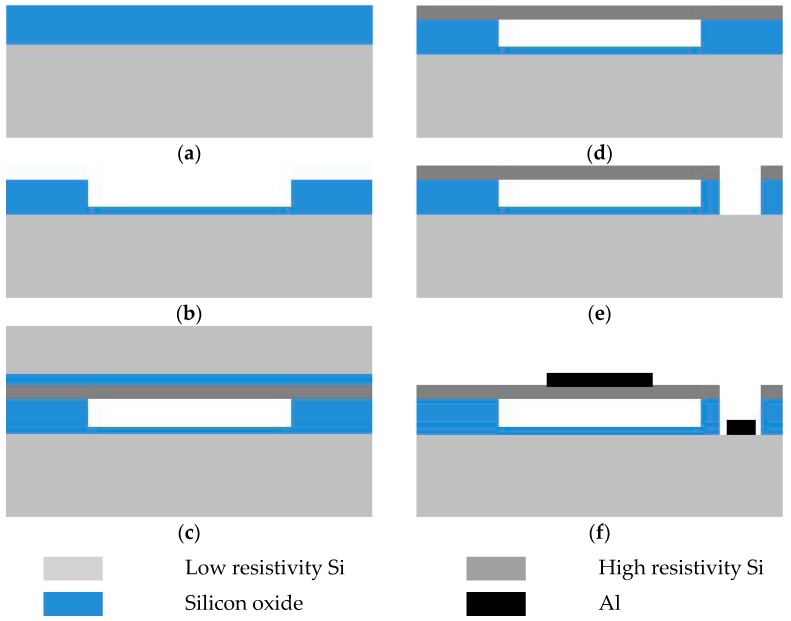
Process flow of micromachined ultrasonic transducer (CMUT) fabrication. (**a**) Thermal oxidation; (**b**) Dry etching for cavity; (**c**) Wafer bonding for the substrate with silicon on insulator (SOI); (**d**) Removing the silicon substrate and the buried oxide of the SOI; (**e**) Silicon and oxide etching for ground contact; (**f**) Aluminum deposition for the top and bottom electrodes.

The process flow to fabricate a CMUT is shown in Figure 5. Each cell was designed to be a circle shape with a 60-μm-radius membrane and all are electrically connected in parallel. Fabrication began on a 4 in (100) conductive silicon wafer covered with a 800 nm thermal silicon dioxide layer, as illustrated in Figure 5a. The silicon wafer was doped to achieve high conduction, which was used as the bottom electrode in the CMUT. Then the cavity was formed by patterning the thermal silicon oxide layer, as shown in Figure 5b. We used an SOI wafer with a device layer thickness of 2 μm, which defined the membrane thickness. The device layer of the SOI is made of single crystal silicon, which has low internal stress and well-defined material parameters: Young’s modulus (*E*), Poisson’s ratio (σ) and density (ρ). The SOI wafer was bonded to the substrate with the goal to transfer the device layer to the substrate. Before wafer direct bonding, both the SOI wafer and processed substrate were treated by wet chemical and O_2_ plasma activations. The wafers were first cleaned by acetone, ethanol, and H_2_SO_4_/H_2_O_2_ solutions followed by a de-ionized (DI) water rinse. Next, modified RCA1 and RCA2 solutions were used to process the wafers for 30 min at 70–80 °C. After rinsing in DI water, the wafers were immersed in an HNO_3_/H_2_O/HF mixture for about 3 min at 70–80 °C followed by rinsing in DI water and blow drying. Thereafter, both wafers were activated by O_2_ plasma at 200 W for 60 s.

After the wet chemical and O_2_ plasma activations, both wafers were brought into contact and annealed in a commercial wafer bonder (Suss SB6e) at 400 °C with 3000 mbar pressure for 2 h in vacuum (<10^−4^ mbar), as indicated in Figure 5c. After completion of the bonding process, the handle layer of the bonded SOI wafer was removed by a tetramethylammonium hydroxide (TMAH) solution at 80 °C. In this step, the buried oxide layer provided a TMAH etch stop. The buried oxide layer was then removed in a buffered hydrofluoric acid (BHF) solution that stopped on the silicon surface, as shown in Figure 5d. This completed the membrane fabrication. Subsequently, the membrane was patterned by the photolithography and dry etching step to open a hole through the top layer to contact the bottom electrode of the CMUT, as illustrated in Figure 5e. For electrical connection, aluminum was sputtered by a metal sputter system and patterned to serve as the top and bottom electrodes by lift-off technology, as shown in Figure 5f.

**Figure 6:**
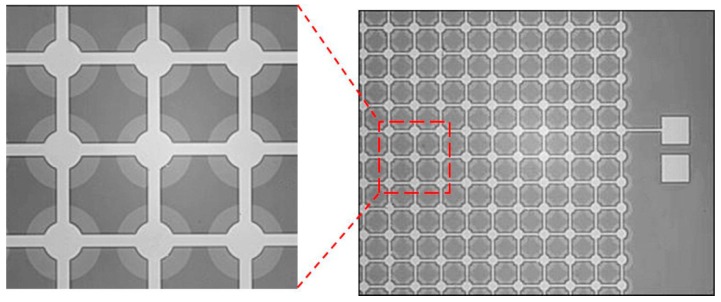
The infrared images of CMUT array.

**Figure 7:**
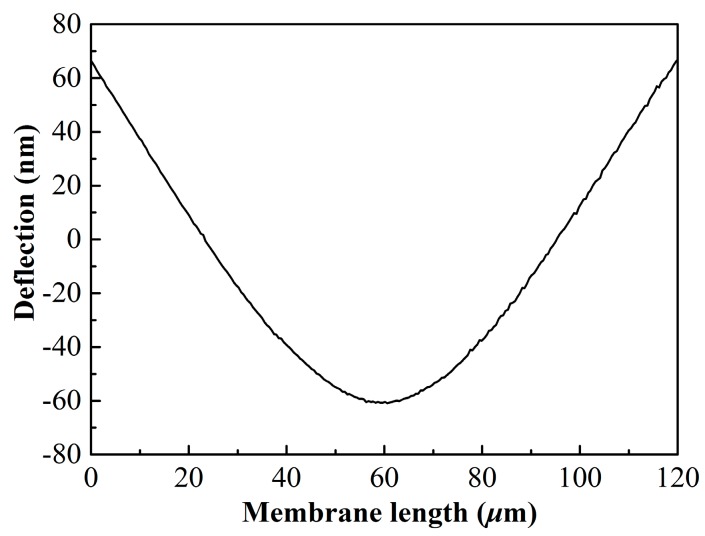
Static deflection profile measured along the CMUT width though the center of the membrane under atmospheric pressure.

In order to inspect the bonding interface under the membrane, the designed devices were observed by an infrared imaging system. It can be seen that there is no damage or falling off of the membranes and electrodes, as shown in Figure 6. No voids or holes are formed in the bonding area. The wafers are strongly bonded to withstand the subsequent removal of the SOI handle layer and buried oxide layer in the wet etching processes. Because the cavity beneath the membrane was considered vacuum-sealed, the membrane deflected under atmospheric pressure. We measured the static deflection along the CMUT width though the center of the membrane in the atmospheric environment by AFM, as shown in Figure 7. A center displacement of 125.15 nm is observed as expected, which indicates good vacuum sealing of the cavity. These results demonstrate the good bonding quality and prove the feasibility of the low-temperature fabrication process of CMUTs.

## 4. Device Characterizations 

**Figure 8:**
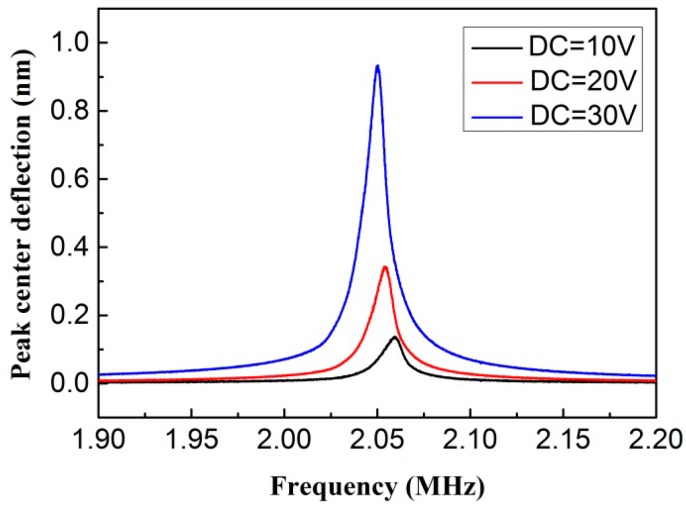
Peak center deflection as a function of frequency for CMUT at different DC levels with an AC voltage of 1 V_pp_ in air.

In order to verify the functionality and properties of the CMUT, the fabricated device was characterized by electrical and acoustic measurements. We measured the electromechanical response of the CMUT using a Polytec laser vibrometer. In this mode, the resonance frequency of 2.06 MHz was obtained without the DC bias voltage. When applying the DC bias voltage from 10 to 30 V with an AC voltage of 1 V_pp_ between the electrodes, the peak center deflection as a function of frequency is shown in Figure 8. The excitation frequency was tuned from 1.90 to 2.20 MHz in order to detect the resonance frequency, at which the peak center deflection was maximized. For the 30 V DC bias voltage, the −3 dB bandwidth is 11 kHz, leading to the *Q*-factor of 187. The resonant peak shifts towards lower frequencies with the increase of the DC bias voltage, while the vibration amplitude increases significantly. This phenomenon is caused by the nonlinearity of electromechanical interaction, and is called the spring softening effect which is common to all MEMS devices actuated with electrostatic forces.

The basic building block of the CMUT resonator is a circular plate, which resonates in the first flexural mode. The dimensions and the material properties of the circular plate determine the resonant frequency *f*_0_, as:

(1) $$f_{0}=0.47\frac{t}{a^{2}}\sqrt{\frac{E}{\rho (1-\nu^{2})}}$$

where ρ, *a*, *t*, *E* and *v* are the density, radius, thickness, Young’s modulus and Poisson’s ratio of the membrane, respectively [20]. Based on this equation, the theoretical resonance frequency was 2.03 MHz. It can be observed the measured resonant frequency is in good agreement with the theoretical result, indicating that the stresses in the membrane and the fabrication process are well-controlled. There was a difference of only approximately 1% between the measured resonant frequency and the theoretical value. As for the device structure, the membrane thickness has a significant impact on the resonance frequency of the device. Any membrane thickness deviations from the theoretical value could result in a variation in the measured frequency. Additionally, there may be discrepancies between the design and fabricated dimensions of the membranes in the lithography and etching process, which could also lead to disparities of the frequencies.

**Figure 9:**
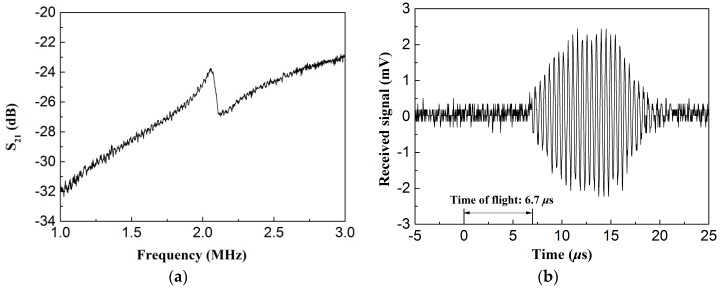
(**a**) Electrical insertion loss (*S*_21_) for CMUT; (**b**) Received signal actuated by 20 cycle burst sine signal.

In addition, the electrical insertion loss (*S*_21_) of the CMUT transducer was measured in air. A vector network analyzer (E5071C, Agilent Technologies, Palo Alto, CA, USA) and a DC supply (U8032A, Agilent Technologies, Palo Alto, CA, USA) were used. The vector network analyzer was connected to the CMUT through a capacitor that blocked the DC voltage, but allowed the AC signal to pass. The DC bias voltage was supplied through a resistor to provide a DC voltage across the transducer. For a 10 V DC bias voltage and 0.2 V AC voltage, a resonance peak at about 2.06 MHz is observed and the *S*_21_ is −24 dB as shown in Figure 9a. In order to further assess the acoustic transmission characterization of CMUTs, we tested the transducer in a water tank with a needle hydrophone (PT-1609344, Institute of Acoustics, UCAS, Beijing, China). The transducer was 0.25 cm^2^ to increase the acoustic pressure. It was excited with 20 cycle burst sine signal of 20 V_pp_ at 2.06 MHz. The hydrophone was placed at the center line of the CMUTs with a distance of 10 mm to receive the sound wave, as seen in Figure 9b. The sound pressure of the transducer is 4.3 kPa with the 0.6 μV/Pa receive sensitivity of the hydrophone at 2.06 MHz. A 6.7 μs time of flight was measured by comparing the time elapsed between the actuated signal and the received signal. Using the traveled distance and time of flight, the velocity of the ultrasonic wave can be calculated to be 1493 m/s in accordance with the well-known speed of sound in water, which is preliminary evidence of the acoustic properties of CMUTs.

For the performance of CMUTs made in the way we describe, the *Q*-factor is 187, which is higher than that presented in [21] (*Q*-factor of 31.5) or [22] (*Q*-factor of 150). In addition, the difference between the measured resonant frequency and the theoretical resonant frequency is only about 1%. This value is smaller than that of the CMUTs achieved in [23], which has a difference of about 6% between these two values. Thirdly, the electrical insertion loss (*S*_21_) is about −24 dB, which is better than that of −42 dB presented in [24]. Finally, we measured the acoustic pressure of 4.3 kPa for 256 cells. As shown in [25], the acoustic pressure is 8.2 kPa for 2708 cells. These measurements indicate that CMUTs made in the way we describe have a good performance.

## 5. Conclusions

In this paper, CMUTs have been successfully fabricated by the wafer direct bonding process at 400 °C. Our studies have focused on activation methods in order to satisfy this temperature specification. These studies indicated that using the wet chemical and O_2_ plasma treatment simultaneously has good activation effects to achieve the low-temperature wafer direct bonding. The surface roughness and contact angle were investigated to acquire an optimized bonding process. The contact angle of 0° was obtained by this process. The surface roughness decreased with the O_2_ plasma activation time until 60 s and then increased significantly, which indicated the activation time of 60 s was suitable for bonding. The infrared images and static deflection at atmospheric pressure have been evaluated to prove the good bonding effect.

To verify the validity of the process and to determine the properties of the CMUTs, the device was characterized by electrical and acoustic measurements. The resonance frequency of the CMUT at 2.06 MHz in air was measured. A spring softening effect was clearly observed, exhibiting expected characteristics. The measured electrical insertion loss of the CMUT was presented. The transducers were tested in water in order to determine the CMUT acoustic transmission behavior. These analyses demonstrated the functionality of the implemented CMUTs fabricated by the low-temperature process. This low-temperature fabrication technology will enable the integration of CMUTs directly above integrated circuit (IC) for feasible, highly compact ultrasonic systems, resulting in a smaller size, lower power and higher performance.

## Figures and Tables

**Table 1:** The parameters of the designed capacitive micromachined ultrasonic transducer (CMUT) structure.

Parameters	Value
Membrane radius (μm)	60
Membrane thickness (μm)	2
Electrode radius (μm)	30
Electrode thickness (μm)	0.3
Number of cells	256
Insulation layer thickness (μm)	0.2
Cavity depth (μm)	0.6
